# Tetrakis(thiourea-κ*S*)palladium(II) dithio­cyanate

**DOI:** 10.1107/S160053680801088X

**Published:** 2008-04-23

**Authors:** Shafqat Nadeem, M. Khawar Rauf, Masahiro Ebihara, Syed Ahmed Tirmizi, Saeed Ahmad

**Affiliations:** a Department of Chemistry, Quaid-i-Azam University, Islamabad 45320, Pakistan; b Department of Chemistry, Faculty of Engineering, Gifu University, Yanagido, Gifu 501-1193, Japan; c Department of Chemistry, University of Engineering and Technology, Lahore 54890, Pakistan

## Abstract

The title compound, [Pd(CH_4_N_2_S)_4_](SCN)_2_, consists of complex [Pd(TU)_4_]^2+^ [TU = thio­urea, SC(NH_2_)_2_] cations and thio­cyanate counter-anions. The Pd^II^ cation is situated on an inversion centre and exhibits an almost square-planar coordination by the S atoms of the TU ligands. The complex cations are connected through the thio­cyanate ions *via* N—H⋯N [2.922 (3)–3.056 (3) Å] and N—H⋯S [3.369 (2)–3.645 (2) Å] hydrogen bonds.

## Related literature

For the coordination chemistry of thio­nes and thio­nates, and for biomolecules possessing thio­amido binding sites, see: Akrivos (2001 ▶); Raper (1996 ▶); Cusumano *et al.* (2005 ▶). For other structures listed in the Cambridge Structural Database (Allen, 2002 ▶) that contain transition metals and thio­urea ligands, see: Bott *et al.* (1998 ▶); Dupa & Krebs (1973 ▶); Gale *et al.* (2006 ▶); Hunt *et al.* (1979 ▶); Taylor *et al.* (1974 ▶).
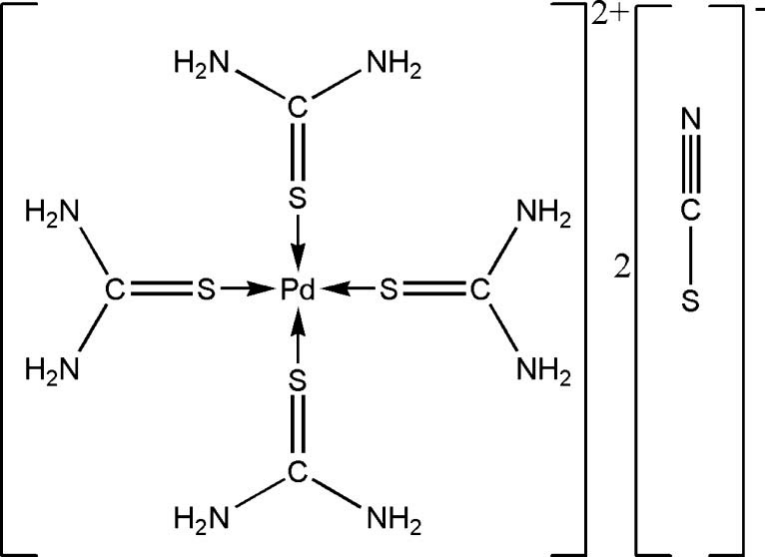


## Experimental

### 

#### Crystal data

[Pd(CH_4_N_2_S)_4_](SCN)_2_
*M* *_r_* = 527.05
Monoclinic, 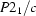

*a* = 8.136 (3) Å
*b* = 12.966 (5) Å
*c* = 8.810 (3) Å
β = 91.12 (5)°
*V* = 929.3 (6) Å^3^
*Z* = 2
Mo *K*α radiation
μ = 1.69 mm^−1^
*T* = 123 (2) K
0.30 × 0.25 × 0.22 mm

#### Data collection

Rigaku/MSC Mercury CCD diffractometer
Absorption correction: integration (*NUMABS*; Higashi, 1999 ▶) *T*_min_ = 0.632, *T*_max_ = 0.708
7301 measured reflections
2117 independent reflections
2040 reflections with *I* > 2σ(*I*)
*R*_int_ = 0.025

#### Refinement

*R*[*F* ^2^ > 2σ(*F* ^2^)] = 0.022
*wR*(*F* ^2^) = 0.043
*S* = 1.35
2117 reflections
106 parameters
H-atom parameters constrained
Δρ_max_ = 0.66 e Å^−3^
Δρ_min_ = −0.48 e Å^−3^

### 

Data collection: *CrystalClear* (Molecular Structure Corporation & Rigaku, 2001 ▶); cell refinement: *CrystalClear*; data reduction: *TEXSAN* (Molecular Structure Corporation & Rigaku, 2004 ▶); program(s) used to solve structure: *SIR97* (Altomare *et al.*, 1999 ▶); program(s) used to refine structure: *SHELXL97* (Sheldrick, 2008 ▶); molecular graphics: *ORTEPII* (Johnson, 1976 ▶); software used to prepare material for publication: *SHELXL97* and *TEXSAN*.

## Supplementary Material

Crystal structure: contains datablocks I, global. DOI: 10.1107/S160053680801088X/wm2176sup1.cif

Structure factors: contains datablocks I. DOI: 10.1107/S160053680801088X/wm2176Isup2.hkl

Additional supplementary materials:  crystallographic information; 3D view; checkCIF report

## Figures and Tables

**Table:** 

Pd1—S2	2.3302 (11)
Pd1—S1	2.3448 (8)

**Table:** 

S2—Pd1—S2^i^	180
S2—Pd1—S1	87.86 (3)
S2^i^—Pd1—S1	92.14 (3)

Symmetry code: (i) 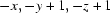.

**Table 2:** Hydrogen-bond geometry (Å, °)

*D*—H⋯*A*	*D*—H	H⋯*A*	*D*⋯*A*	*D*—H⋯*A*
N1—H1*B*⋯S3	0.88	2.58	3.369 (2)	150
N1—H1*A*⋯S2^ii^	0.88	2.60	3.466 (2)	166
N2—H2*A*⋯S3^iii^	0.88	2.78	3.615 (2)	158
N2—H2*B*⋯N5^iv^	0.88	2.04	2.922 (3)	178
N3—H3*B*⋯S3	0.88	2.82	3.645 (2)	157
N3—H3*A*⋯S1^v^	0.88	2.73	3.531 (2)	153
N4—H4*B*⋯S3^vi^	0.88	2.61	3.482 (2)	173
N4—H4*A*⋯N5^vii^	0.88	2.50	3.056 (3)	121

Symmetry codes: (ii) 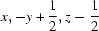; (iii) 
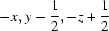
; (iv) 
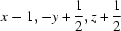
; (v) 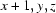; (vi) 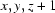; (vii) 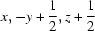.

**Fig. 1.**
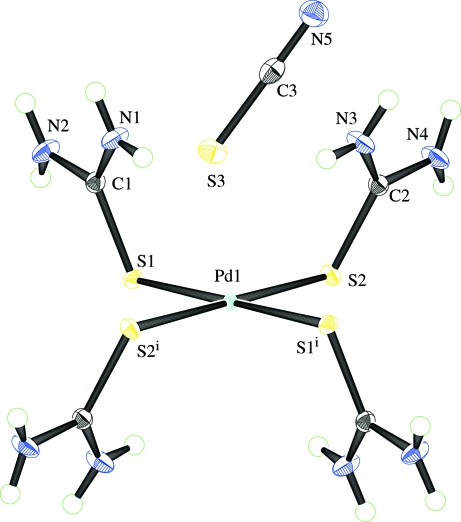
Molecular structure of (I) showing the atom-labelling scheme and displacement ellipsoids at the 30% probability level. Unlabelled atoms and atoms labelled by superscript i) are related by the symmetry operator i) 1-x, y, z.

## supplementary crystallographic information

### Comment

Thiourea (TU), SC(NH_2_)_2_, is a simple ambidentate ligand capable of binding to transition metals *via* the sulfur or the nitrogen atoms. Complex formation with such ligands provides model systems for the interaction of naturally occurring biomolecules possessing thioamido binding sites (Akrivos, 2001; Raper, 1996; Cusumano *et al.*, 2005). The ability of TU to form stable adducts with a variety of transition metals, *e.g.* Cu, Ag, Au and Pt, is well established. The crystal structures of several such complexes have been determined (Bott *et al.*, 1998; Gale *et al.*, 2006). These studies demonstrate that TU can act both as a terminal ligand in monomeric complexes (Hunt *et al.*, 1979), or as a bridging ligand in polymeric complexes (Taylor *et al.*, 1974). In order to investigate other transition metal complexes of thiourea, we report here the crystal structure of a monomeric complex, viz. [Pd(SC(NH_2_)_2_)_4_](SCN)_2_, (I).

The crystal structure of (I) is composed of complex [Pd(TU)_4_]^+2^ cations and thiocyanate counter anions. The Pd^2+^ ion is situated on an inversion centre and, as expected for a *d*^8^ system, has an almost square planar environment with *cis* angles (S—Pd—S) ranging from 87.87 (2) to 92.13 (2)°, and *trans* angles (S—Pd—S) of 180.0°. The TU ligands are coordinated to Pd^II^ at almost equal distances. The Pd—S bond lengths of 2.3302 (8) and 2.3448 (7) Å (Table 1) are comparable to those of similar compounds reported in the literature (Gale *et al.*, 2006). In the cationic complex, TU ligands behave as S–donors and all four ligands are binding in a terminal mode. Therefore no bridging of metal centers are found as it is observed in some other metal-thiourea compounds, for example, [Cu_4_(TU)_7_(SO_4_)_2_]NO_3_ (Bott *et al.*, 1998) and [Ag_2_(TU)_6_](ClO_4_)_2_ (Dupa & Krebs, 1973). The C—S and C—N bond lengths of  1.723 (2) Å and 1.326 (3) Å, respectively, agree with those of coordinated thiourea molecules reported in the Cambridge Crystallographic database (Allen, 2002). In the crystal structure, the building units are connected *via* hydrogen bonds of the type N—H···N [2.922 (3)–3.058 (3) Å] and N—H···S [3.370 (2)–3.646 (2) Å] (see Table 2).

### Experimental

Crystals of (I) were obtained by adding 4 equivalents of thiourea in 15 ml methanol to a solution of K_2_[PdCl_2_] (0.326 g) in 15 ml of water and stirring for one h. The resulting orange solution was kept after filtration at room temperature for three d. Orange crystals of (I) were obtained on slow evaporation. The counter anion SCN^-^ has apparently been introduced due to impurities (presumably KSCN), that were present in thiourea.

### Refinement

The H atoms attached to the N atoms were placed in idealized positions and refined with a N—H distance of 0.88 Å and *U*_iso_(H) = 1.2*U*_eq_(N).

### Crystal data

**Table:**

[Pd(CH_4_N_2_S)_4_](SCN)_2_	*F*_000_ = 528
*M**_r_* = 527.05	*D*_x_ = 1.884 Mg m^−^^3^
Monoclinic, *P*2_1_/*c*	Mo *K*α radiation λ = 0.71070 Å
Hall symbol: -P 2ybc	Cell parameters from 3103 reflections
*a* = 8.136 (3) Å	θ = 3.4–27.5º
*b* = 12.966 (5) Å	µ = 1.69 mm^−^^1^
*c* = 8.810 (3) Å	*T* = 123 (2) K
β = 91.12 (5)º	Prism, orange
*V* = 929.3 (6) Å^3^	0.30 × 0.25 × 0.22 mm
*Z* = 2	

### Data collection

**Table:**

Rigaku/MSC Mercury CCD diffractometer	2117 independent reflections
Radiation source: fine-focus sealed tube	2040 reflections with *I* > 2σ(*I*)
Monochromator: graphite	*R*_int_ = 0.025
*T* = 123(2) K	θ_max_ = 27.5º
ω scans	θ_min_ = 3.9º
Absorption correction: integration(NUMABS; Higashi, 1999)	*h* = −8→10
*T*_min_ = 0.632, *T*_max_ = 0.708	*k* = −16→13
7301 measured reflections	*l* = −11→11

### Refinement

**Table:**

Refinement on *F*^2^	Secondary atom site location: difference Fourier map
Least-squares matrix: full	Hydrogen site location: inferred from neighbouring sites
*R*[*F*^2^ > 2σ(*F*^2^)] = 0.022	H-atom parameters constrained
*wR*(*F*^2^) = 0.043	*w* = 1/[σ^2^(*F*_o_^2^) + 0.6382*P*] where *P* = (*F*_o_^2^ + 2*F*_c_^2^)/3
*S* = 1.35	(Δ/σ)_max_ = 0.001
2117 reflections	Δρ_max_ = 0.66 e Å^−^^3^
106 parameters	Δρ_min_ = −0.48 e Å^−^^3^
Primary atom site location: structure-invariant direct methods	Extinction correction: none

### Special details

**Table:**

Geometry. All e.s.d.'s (except the e.s.d. in the dihedral angle between two l.s. planes) are estimated using the full covariance matrix. The cell e.s.d.'s are taken into account individually in the estimation of e.s.d.'s in distances, angles and torsion angles; correlations between e.s.d.'s in cell parameters are only used when they are defined by crystal symmetry. An approximate (isotropic) treatment of cell e.s.d.'s is used for estimating e.s.d.'s involving l.s. planes.
Refinement. Refinement of *F*^2^ against ALL reflections. The weighted *R*-factor *wR* and goodness of fit *S* are based on *F*^2^, conventional *R*-factors *R* are based on *F*, with *F* set to zero for negative *F*^2^. The threshold expression of *F*^2^ > σ(*F*^2^) is used only for calculating *R*-factors(gt) *etc*. and is not relevant to the choice of reflections for refinement. *R*-factors based on *F*^2^ are statistically about twice as large as those based on *F*, and *R*- factors based on ALL data will be even larger.

### Fractional atomic coordinates and isotropic or equivalent isotropic displacement parameters (Å^2^)

**Table:**

	*x*	*y*	*z*	*U*_iso_*/*U*_eq_	
Pd1	0.0000	0.5000	0.5000	0.00900 (6)	
S1	−0.19061 (6)	0.37251 (3)	0.56476 (5)	0.01265 (10)	
C1	−0.1354 (2)	0.26304 (14)	0.4664 (2)	0.0140 (4)	
N1	−0.0189 (2)	0.26297 (13)	0.36425 (19)	0.0197 (4)	
H1A	0.0068	0.2054	0.3175	0.024*	
H1B	0.0332	0.3205	0.3429	0.024*	
N2	−0.2134 (2)	0.17612 (13)	0.49809 (19)	0.0199 (4)	
H2A	−0.1874	0.1187	0.4511	0.024*	
H2B	−0.2911	0.1759	0.5661	0.024*	
S2	0.13159 (6)	0.47003 (4)	0.73312 (5)	0.01300 (10)	
C2	0.3204 (2)	0.41505 (14)	0.7012 (2)	0.0141 (4)	
N3	0.3774 (2)	0.39827 (14)	0.56415 (18)	0.0201 (4)	
H3A	0.4743	0.3693	0.5534	0.024*	
H3B	0.3184	0.4160	0.4835	0.024*	
N4	0.4105 (2)	0.38793 (14)	0.82155 (19)	0.0219 (4)	
H4A	0.5073	0.3591	0.8096	0.026*	
H4B	0.3736	0.3988	0.9134	0.026*	
S3	0.22933 (7)	0.42269 (4)	0.17265 (6)	0.02044 (12)	
C3	0.4076 (3)	0.36611 (15)	0.2055 (2)	0.0186 (4)	
N5	0.5334 (2)	0.32525 (15)	0.2287 (2)	0.0260 (4)	

### Atomic displacement parameters (Å^2^)

**Table:**

	*U*^11^	*U*^22^	*U*^33^	*U*^12^	*U*^13^	*U*^23^
Pd1	0.00878 (10)	0.00771 (9)	0.01049 (9)	0.00000 (7)	−0.00061 (7)	0.00047 (7)
S1	0.0121 (2)	0.0103 (2)	0.0156 (2)	−0.00127 (17)	0.00147 (17)	−0.00054 (16)
C1	0.0152 (10)	0.0128 (9)	0.0137 (9)	−0.0004 (7)	−0.0027 (7)	0.0001 (7)
N1	0.0228 (10)	0.0129 (8)	0.0238 (9)	−0.0030 (7)	0.0077 (7)	−0.0057 (6)
N2	0.0248 (10)	0.0115 (8)	0.0236 (9)	−0.0039 (7)	0.0073 (7)	−0.0027 (7)
S2	0.0111 (2)	0.0163 (2)	0.0115 (2)	0.00134 (17)	−0.00052 (16)	0.00123 (16)
C2	0.0126 (9)	0.0124 (9)	0.0172 (9)	−0.0010 (7)	−0.0007 (7)	0.0011 (7)
N3	0.0166 (9)	0.0275 (9)	0.0162 (8)	0.0096 (7)	0.0005 (7)	0.0019 (7)
N4	0.0170 (9)	0.0309 (10)	0.0176 (8)	0.0108 (8)	−0.0040 (7)	0.0005 (7)
S3	0.0193 (3)	0.0202 (2)	0.0220 (2)	0.0026 (2)	0.0054 (2)	0.00404 (19)
C3	0.0235 (12)	0.0183 (10)	0.0144 (9)	−0.0049 (8)	0.0061 (8)	−0.0029 (7)
N5	0.0232 (11)	0.0256 (9)	0.0292 (10)	0.0018 (8)	0.0031 (8)	−0.0007 (8)

### Geometric parameters (Å, °)

**Table:**

Pd1—S2	2.3302 (11)	N2—H2B	0.8800
Pd1—S2^i^	2.3302 (11)	S2—C2	1.721 (2)
Pd1—S1	2.3448 (8)	C2—N3	1.320 (3)
Pd1—S1^i^	2.3448 (8)	C2—N4	1.325 (3)
S1—C1	1.727 (2)	N3—H3A	0.8800
C1—N1	1.320 (3)	N3—H3B	0.8800
C1—N2	1.326 (3)	N4—H4A	0.8800
N1—H1A	0.8800	N4—H4B	0.8800
N1—H1B	0.8800	S3—C3	1.646 (2)
N2—H2A	0.8800	C3—N5	1.167 (3)
			
S2—Pd1—S2^i^	180.0	C1—N2—H2B	120.0
S2—Pd1—S1	87.86 (3)	H2A—N2—H2B	120.0
S2^i^—Pd1—S1	92.14 (3)	C2—S2—Pd1	108.72 (7)
S2—Pd1—S1^i^	92.14 (3)	N3—C2—N4	119.29 (18)
S2^i^—Pd1—S1^i^	87.86 (3)	N3—C2—S2	123.27 (15)
S1—Pd1—S1^i^	180.0	N4—C2—S2	117.44 (15)
C1—S1—Pd1	106.11 (7)	C2—N3—H3A	120.0
N1—C1—N2	119.74 (17)	C2—N3—H3B	120.0
N1—C1—S1	122.68 (15)	H3A—N3—H3B	120.0
N2—C1—S1	117.57 (15)	C2—N4—H4A	120.0
C1—N1—H1A	120.0	C2—N4—H4B	120.0
C1—N1—H1B	120.0	H4A—N4—H4B	120.0
H1A—N1—H1B	120.0	N5—C3—S3	179.5 (2)
C1—N2—H2A	120.0		
			
S2—Pd1—S1—C1	−101.78 (7)	S1—Pd1—S2—C2	112.12 (7)
S2^i^—Pd1—S1—C1	78.22 (7)	S1^i^—Pd1—S2—C2	−67.88 (7)
Pd1—S1—C1—N1	−6.95 (19)	Pd1—S2—C2—N3	2.44 (18)
Pd1—S1—C1—N2	172.15 (14)	Pd1—S2—C2—N4	−176.95 (14)

Symmetry codes: (i) −*x*, −*y*+1, −*z*+1.

### Hydrogen-bond geometry (Å, °)

**Table:**

*D*—H···*A*	*D*—H	H···*A*	*D*···*A*	*D*—H···*A*
N1—H1B···S3	0.88	2.58	3.369 (2)	150
N1—H1A···S2^ii^	0.88	2.60	3.466 (2)	166
N2—H2A···S3^iii^	0.88	2.78	3.615 (2)	158
N2—H2B···N5^iv^	0.88	2.04	2.922 (3)	178
N3—H3B···S3	0.88	2.82	3.645 (2)	157
N3—H3A···S1^v^	0.88	2.73	3.531 (2)	153
N4—H4B···S3^vi^	0.88	2.61	3.482 (2)	173
N4—H4A···N5^vii^	0.88	2.50	3.056 (3)	121

Symmetry codes: (ii) *x*, −*y*+1/2, *z*−1/2; (iii) −*x*, *y*−1/2, −*z*+1/2; (iv) *x*−1, −*y*+1/2, *z*+1/2; (v) *x*+1, *y*, *z*; (vi) *x*, *y*, *z*+1; (vii) *x*, −*y*+1/2, *z*+1/2.
